# Mesenchymal Stem Cells Protect Nucleus Pulposus Cells from Compression-Induced Apoptosis by Inhibiting the Mitochondrial Pathway

**DOI:** 10.1155/2017/9843120

**Published:** 2017-12-14

**Authors:** Sheng Chen, Lei Zhao, Xiangyu Deng, Deyao Shi, Fashuai Wu, Hang Liang, Donghua Huang, Zengwu Shao

**Affiliations:** Department of Orthopaedic Surgery, Union Hospital, Tongji Medical College, Huazhong University of Science and Technology, Wuhan 430022, China

## Abstract

**Objective:**

Excessive apoptosis of nucleus pulposus cells (NPCs) induced by various stresses, including compression, contributes to the development of intervertebral disc degeneration (IVDD). Mesenchymal stem cells (MSCs) can benefit the regeneration of NPCs and delay IVDD, but the underlying molecular mechanism is poorly understood. This study aimed to evaluate the antiapoptosis effects of bone marrow-derived MSC (BMSC) on rat NPCs exposed to compression and investigate whether the mitochondrial pathway was involved.

**Methods:**

BMSCs and NPCs were cocultured in the compression apparatus at 1.0 MPa for 36 h. Cell viability, apoptosis, mitochondrial function, and the expression of apoptosis-related proteins were evaluated.

**Results:**

The results showed that coculturing with BMSCs increased the cell viability and reduced apoptosis of NPCs exposed to compression. Meanwhile, BMSCs could relieve the compression-induced mitochondrial damage of NPCs by decreasing reactive oxygen species level and maintaining mitochondrial membrane potential as well as mitochondrial integrity. Furthermore, coculturing with BMSCs suppressed the activated caspase-3 and activated caspase-9, decreased the expressions of cytosolic cytochrome *c* and Bax, and increased the expression of Bcl-2.

**Conclusions:**

Our results suggest that BMSCs can protect against compression-induced apoptosis of NPCs by inhibiting the mitochondrial pathway and thus enhance our understanding on the MSC-based therapy for IVDD.

## 1. Introduction

Intervertebral disc degeneration (IVDD) is the main cause of low back pain (LBP) with high prevalence, which leads to disability and creates heavy financial burden globally [1–3]. The intervertebral disc (IVD) is composed of three parts: nucleus pulposus (NP), annulus fibrous (AF), and cartilaginous endplates. The centrally situated NP consists of NP cells (NPCs) and extracellular matrix (ECM), and the outer AF is mainly made of collagen fibers. Evidences show that the IVD progressively degenerates with the number of NPC loss, ECM reduction, and type I collagen synthesis increase [4]. And recently, many studies in vitro and in vivo have indicated that excessive apoptosis of NPCs induced by various stresses, including compression, hypoxia or reactive oxygen species (ROS), plays an essential role in the progression of IVDD [5–7].

The IVD functions as a shock absorber, and external forces on the spine lead to intense stresses that act on the IVD. From a mechanical point of view, disc cells embedded in the different areas are exposed to wide ranges of mechanical loads [8, 9]. Inappropriate or excessive compressive force stimulus applied to intervertebral discs (IVDs) is an important contributing factor in causing disc degeneration. Previous studies have suggested that excessive loading affects the synthesis of ECM and promotes the secretion of inflammatory factors in NPCs [10, 11]. We have reported that apoptosis could be induced by compression at a magnitude of 1 MPa via mitochondrial or intrinsic pathways in rabbit NPCs previously [12]. However, there are few researches to study how to reverse the apoptosis of NPCs induced by compression and thus the repair of IVDD.

Mesenchymal stem cells (MSCs), especially bone marrow-derived MSC- (BMSC-) based therapies, have been commonly used in IVDD repair and have shown exciting perspectives [13, 14]. A great number of studies have discussed the interaction between MSCs and NPCs under different conditions. It was reported that coculture of MSCs and NPCs facilitated MSC differentiation towards the NP cell phenotype [15, 16] and promoted the synthesis of ECM in degenerated NPCs [15, 17]. However, limited studies demonstrated the antiapoptosis effect of MSCs on NPCs and the specific mechanisms under compression condition. Therefore, this study aimed to evaluate the antiapoptosis effect of BMSCs on rat NPCs under compression and investigate whether the mitochondrial pathway was involved.

## 2. Methods

### 2.1. BMSCs and NPCs Culture

The experimental procedures were approved by the Institutional Animal Care and Use Committee of Tongji Medical College of Huazhong University of Science and Technology. The NPCs were isolated from Sprague-Dawley rats (male, 3 months and 250–300 g) as described previously [18]. The obtained cells were suspended and cultured in Dulbecco's Modified Eagle's Medium/Ham's F-12 (DMEM/F-12, Gibco, USA) containing 10% fetal bovine serum (FBS, Gibco, USA) supplemented with 1% penicillin-streptomycin (Sigma) at 37°C with 5% CO_2_. The media were changed every two days, and the primary culture was 1 : 2 subcultured when cells reached confluence over 80%. The second generation NP cells were used in this study.

We used the same rats to isolate and obtain bone marrow MSCs simultaneously, as previously described [19]. The MSCs were maintained in DMEM/F-12 (Gibco, USA) containing 10% FBS (Gibco, USA) supplemented with 1% penicillin-streptomycin (Sigma) at 37°C with 5% CO_2_. NPCs and BMSCs were from the same source at each coculture system.

### 2.2. Indirect Cocultures and Application of a Compression Apparatus

The indirect cocultures were conducted in 6-well plates with 0.4 *μ*m pore-size transwell inserts. Passage 2 NPCs and passage 3 BMSCs were used. BMSCs were seeded into transwell inserts, whereas NPCs were plated into the lower chamber. Cells were seeded at ratios of 50 : 50 (10 × 10^4^ per well). Cocultured cells were maintained in 10% DMEM/F-12 at 37°C with 5% CO_2_.

To determine the antiapoptosis effect of BMSCs on NPCs exposed to static compression, cocultured cells were cultured in a custom-made compression apparatus as previously described [12, 18]. The coculture system was subjected to 1 MPa compression load (CL) for 36 h. The groups of the experiment were as follows: (i) NPC alone as control; (ii) NPC + BMSC; (iii) NPC + BMSC + CL; and (iv) NPC + CL.

### 2.3. Cell Viability Measurement

Cell viability was measured by CCK-8 (Dojindo, Japan) with modifications. CCK-8 working solution was made by mixing CCK-8 solution and 10% DMEM/F-12 medium at 1 : 9 (*v*/*v*). After different treatments, the culture inserts as well as original culture medium were removed and 2 mL CCK-8 working solution was added. The plates were incubated at 37°C with 5% CO_2_ for 2 h. Then, 100 *μ*L of reacted solution was transferred to a 96-well plate. The surviving cell counts were determined by absorbance detection at 450 nm with a spectrophotometer (BioTek, USA).

### 2.4. Detection of Apoptosis by Flow Cytometry

Apoptosis rate was detected by Annexin V-FITC/PI Apoptosis Detection Kit (KeyGen Biotech, China). In brief, the cells were collected and washed with PBS and then resuspended in 500 *μ*L binding buffer. 5 *μ*L Annexin V-FITC and PI were added and the specimens were incubated in the dark at room temperature for 15 min. The labeled cells were detected via flow cytometry (BD LSR II, Becton Dickinson), and the data were analyzed by FACSDiva Software (Becton Dickinson, USA).

### 2.5. Observation of Cell Morphology

After treatment with compressive stress, cells were observed by inverted microscopy (Olympus, Japan). To further observe the apoptotic features, Hoechst 33258 staining was used. The collected cells were washed with PBS and then stained with Hoechst 33258 (Sigma, USA) for 15 min in the dark according to the manufacturer's instructions. Thereafter, morphologic changes of NPCs were observed and imaged under the inverted fluorescence microscope (Olympus, Japan).

### 2.6. TUNEL Staining

More sensitive TUNEL staining was used to evaluate the cell apoptosis. Following fixation in 4% paraformaldehyde for 1 h at room temperature, the cells were permeabilized with 0.1% TritonX-100 for 10 min. After washed with PBS, the cells were incubated with TUNEL staining (Roche, Germany) for 1 h at 37°C in the dark, according to the manufacturer's protocol. Apoptotic alterations were observed under the inverted fluorescence microscope (Olympus, Japan).

### 2.7. ROS Measurement

The intracellular ROS level was measured by 2,7-dichlorofluorescin diacetate (DCFH-DA; Beyotime, China). In the presence of ROS, DCFH-DA is oxidized into the fluorescent dichlorofluorescein (DCF). After treatment, the collected cells were resuspended in DCFH-DA and incubated in the dark for 30 minutes at 37°C. The mean fluorescence intensity (MFI) of DCF was measured by flow cytometry.

### 2.8. Mitochondrial Membrane Potential (MMP) Assay

MMP was measured by JC-1 (Beyotime, China) according to the manufacturer's instructions. In brief, the harvested NPCs were resuspended in the mixture contained 500 *μ*L 10% DMEM/F-12 and 500 *μ*L JC-1 staining fluid. After incubated in the dark for 30 minutes at 37°C, the cells were washed with ice-cold staining buffer (1x) and resuspended in 500 *μ*L staining buffer (1x). The values of MMP staining expressed as the ratio of red over green fluorescence intensities were determined by flow cytometry.

### 2.9. Transmission Electron Microscopy (TEM)

TEM was performed as previously described [18]. Briefly, harvested cells were washed with PBS and deionized water, respectively, and then pelleted by centrifugation. Cells were prefixed with 2.5% glutaraldehyde for 2 h and postfixed in 1% osmium tetroxide for 2 h. Then the cells were dehydrated in ethanol and infiltrated and embedded in epon 812. Ultrathin sections were stained with uranyl acetate and lead citrate and examined with a Tecnai G^2^ 12 TEM (FEI Company, Holland).

### 2.10. Western Blot Analysis

NPCs were lysed on ice using a standard buffer (Beyotime, China). Total protein was extracted by protein extraction kit (Beyotime, China) and a cell mitochondria Isolation Kit (Beyotime, China) was used to extract the mitochondria-free plasma protein for cytosolic cytochrome *c* detection. The cell lysate was centrifuged at 12,000 ×g for 10 min at 4°C. After protein transfer, the membranes were blocked by nonfat milk and then incubated overnight at 4°C with rat polyclonal antibody against cleaved caspase-3 (Abcam, 1 : 500), Bax (Abcam, 1 : 1000), Bcl-2 (Abcam, 1 : 1000), cleaved caspase-9 (Abcam, 1 : 1000), cytochrome *c* (Abcam, 1 : 1000), and *β*-actin (Abcam, 1 : 3000). After several times of washing, the membrane was incubated with secondary antibodies for 1 h at room temperature. Finally, the immunoreactive membranes were visualized via the enhanced chemiluminescence (ECL) method following the manufacturer's instructions (Amersham Biosciences, USA).

### 2.11. Statistical Analysis

All measurements were performed at least three times. The data were expressed as mean ± standard deviation (SD). Student's *t*-tests were used in the analysis of two-group parameters. One-way analysis of variance (ANOVA) test was used in comparisons of multiple sets of data, followed by the Tukey's post hoc test. *P* < 0.05 were considered significant.

## 3. Results

### 3.1. Coculturing with BMSCs Increased the Cell Viability of NPCs

**Figure 1:**
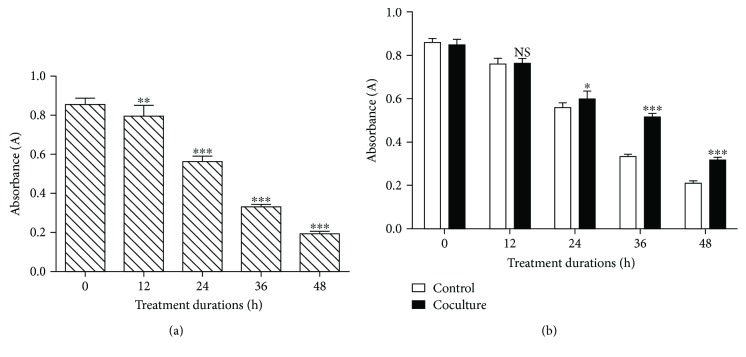
Cell viability of NPCs exposed to compression measuring by the CCK-8 assay. (a) NPCs were exposed to compression for 0, 12, 24, 36, or 48 h. (b) Coculture with BMSCs was applied to all-time point to verify the protective effects of BMSCs. NS means no statistical significant difference. NP cells without compression treatment as control. The data are expressed as mean ± SD from three independent experiments. (^∗^*P* < 0.05, ^∗∗^*P* < 0.01, and ^∗∗∗^*P* < 0.001 versus control).

To determine the effect of BMSCs on the viability of compression-treated NPCs, a CCK-8 assay was performed. As shown in Figure 1(a), the compression inhibited the viability of the NPCs in a time-dependent manner from 0 to 48 h (Figure 1(a), *P* < 0.01). NPCs were divided into coculture and control groups. For NPCs cocultured with BMSCs under compression stress, BMSCs significantly increased the viability of NPCs compared to the cells exposed to compression alone. From the time point 36 h, the *P* value of each group was less than 0.001 (Figure 1(b)). Therefore, the time point 36 h was used in the following experiments.

### 3.2. Protective Effect of BMSCs on Compression-Induced Apoptosis in NPCs

**Figure 2:**
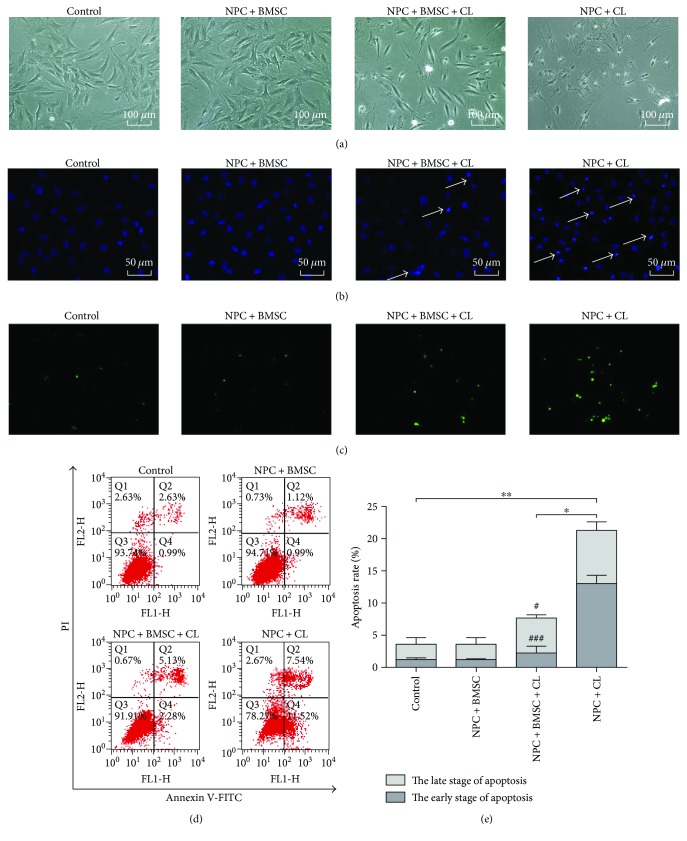
The antiapoptosis effect of BMSCs on NPCs exposed to compression. (a) The phase-contrast photomicrograph of NPCs. (b) Hoechst 33258 staining of NPCs. Apoptotic cells were characterized by the brightly stained condensed nuclei (indicated by arrows). (c) TUNEL staining of NPCs. (d) Representative images of cell apoptosis by flow cytometry analysis after Annexin V/PI dual staining. (e) Summary data showing the apoptosis rate in different groups. The cells at the early stage of apoptosis were stained with Annexin V+/PI−, and the cells at the late stage of apoptosis were stained with Annexin V+/PI+. CL means compression load. The data are expressed as mean ± SD from three independent experiments (^∗^*P* < 0.05 and ^∗∗^*P* < 0.01 versus control or NPC + BMSC + CL; ^#^*P* < 0.05 and ^###^*P* < 0.001 versus NPC + CL).

With compression treatment for 36 h, the NPCs exhibited shrunk or threadlike morphology and almost detached from the plates (Figure 2(a)). Furthermore, Hoechst 33258 staining revealed the brightly stained condensed nuclei, and the number of TUNEL-positive cells increased (Figures 2(b) and 2(c)). As expected, BMSCs could obviously attenuate the morphological changes indicative of apoptosis (Figure 2). The flow cytometry demonstrated that the apoptosis rate of the NPCs treated with compression for 36 h was significantly higher than control (*P* < 0.01). However, coculturing with BMSCs partially prevented this compression-induced apoptosis (Figures 2(d) and 2(e), *P* < 0.05). Interestingly, coculturing with BMSCs mainly reduced the percentage of apoptotic cells at the early stage (Figure 2(e), *P* < 0.001). And there was no significant difference between the control group with the cocultures not exposed to compression.

### 3.3. BMSCs Inhibit Compression-Induced ROS Production

**Figure 3:**
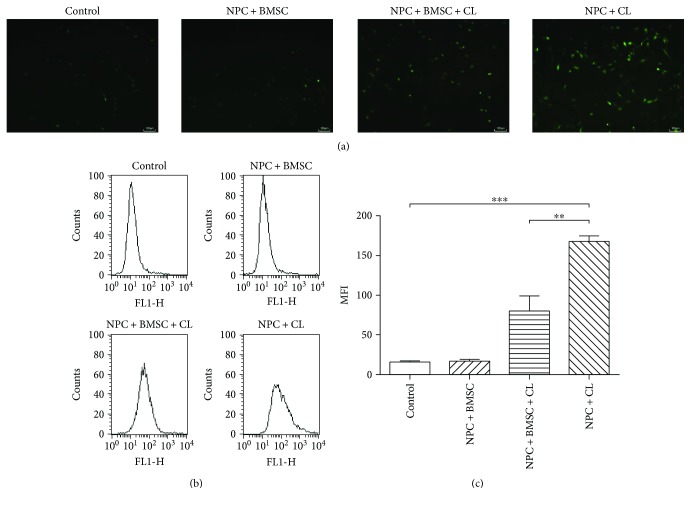
The effect of BMSCs on the compression-induced intracellular accumulation of ROS in NPCs. (a) Typical graphs of ROS imaged by fluorescence microscopy. (b) The intracellular ROS levels were measured by flow cytometry through DCFH-DA staining. (c) Summary data showing the mean fluorescence intensity (MFI) in different groups. CL means compression load. The data are expressed as mean ± SD from three independent experiments (^∗∗^*P* < 0.01 and ^∗∗∗^*P* < 0.001 versus control or NPC + BMSC + CL).

Excessive ROS could impair mitochondrial function and increase the apoptosis rate of NPCs. As shown in the fluorescence images, compression treatment increased the fluorescence intensity, which indicated the production of ROS. Conversely, coculturing with BMSCs reduced the DCF fluorescence induced by compression (Figure 3(a)). Consistent with the fluorescence results, FACS analyses showed that the levels of intracellular ROS production in the NPCs treated with compression increased significantly compared to the untreated control group (*P* < 0.001), whereas coculturing with BMSCs inhibited the compression-induced increase in ROS production (Figures 3(b) and 3(c), *P* < 0.01).

### 3.4. BMSCs Inhibit Compression-Induced Decrease of MMP (Δ*ψ*_m_)

**Figure 4:**
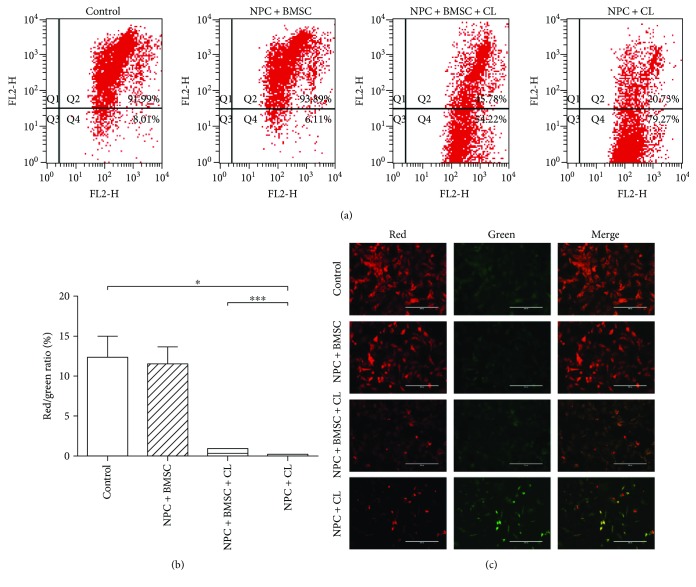
Coculture with BMSCs maintained the MMP of NPCs exposed to compression. (a) The MMP was analyzed by flow cytometry through JC-1 staining. (b) Summary data showing the quantitative MMP expressed as the ratio of red/green fluorescence intensity. (c) The representative fluorescence images of in situ JC-1 staining. CL means compression load. The data are expressed as mean ± SD from three independent experiments (^∗^*P* < 0.05 and ^∗∗∗^*P* < 0.001 versus control or NPC + BMSC + CL).

MMP assay was applied to evaluate mitochondrial function. In the control group, the NPCs stained with JC-1 exhibited intense red fluorescence with weak green fluorescence. But on the contrary, the green fluorescence in the NPCs subjected to compression became stronger, while the red fluorescence became weaker. Coculturing with BMSCs could partly reverse the harmful change and decrease the loss of the Δ*ψ*_m_ (Figure 4(c)). Flow cytometric analysis showed that the NPCs exposed to compression exhibited a remarkable reduction of *Δψ*_m_ compared to controls (*P* < 0.05), which was indicated by the decrease of red/green fluorescence ratio. However, coculturing with BMSCs significantly increased the red/green fluorescence ratio and maintained the Δ*ψ*_m_ of compression-treated NPCs (Figures 4(a) and 4(b), *P* < 0.001).

### 3.5. Observation of the Mitochondrial Ultrastructure of NPCs by TEM

**Figure 5:**
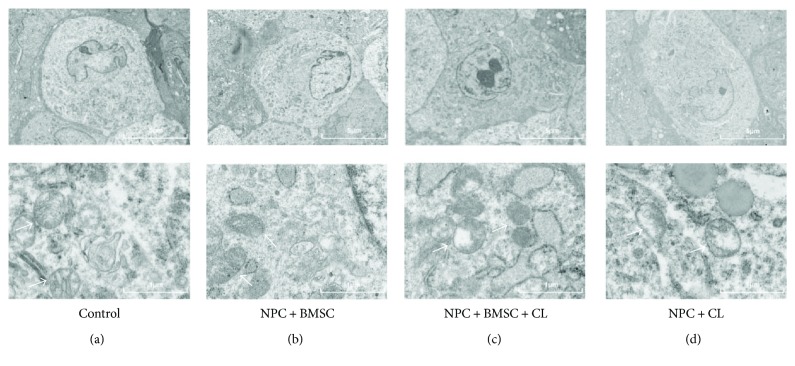
The mitochondrial ultrastructure of NPCs was assessed using TEM. (a) Control group and (b) NPC + BMSC group displayed normal mitochondria. (c) NPC + BMSC + CL group BMSCs improved the ultrastructure collapse of the mitochondria in NPCs with compression treatment. (d) NPC + CL group demonstrated disintegrating cristae and swelling mitochondria. CL means compression load (mitochondria were indicated by arrows).

In order to intuitively observe the mitochondrial ultrastructure of NPCs with compression treatment, TEM was applied to evaluate mitochondrial integrity and state. In the control group, the mitochondrial ultrastructure was normal with well-defined cristae. But in the compression-treated NPCs, disintegrating cristae and swelling mitochondria were observed, which indicated the mitochondria were damaged. Not surprisingly, coculturing with BMSCs exhibited a significant improvement in ultrastructure collapse of the mitochondria. These results suggested that coculturing with BMSCs could improve the mitochondrial state of the NPCs exposed to compression (Figure 5).

### 3.6. Coculturing with BMSCs Blocks Compression-Induced Activation of the Mitochondrial Pathway

**Figure 6:**
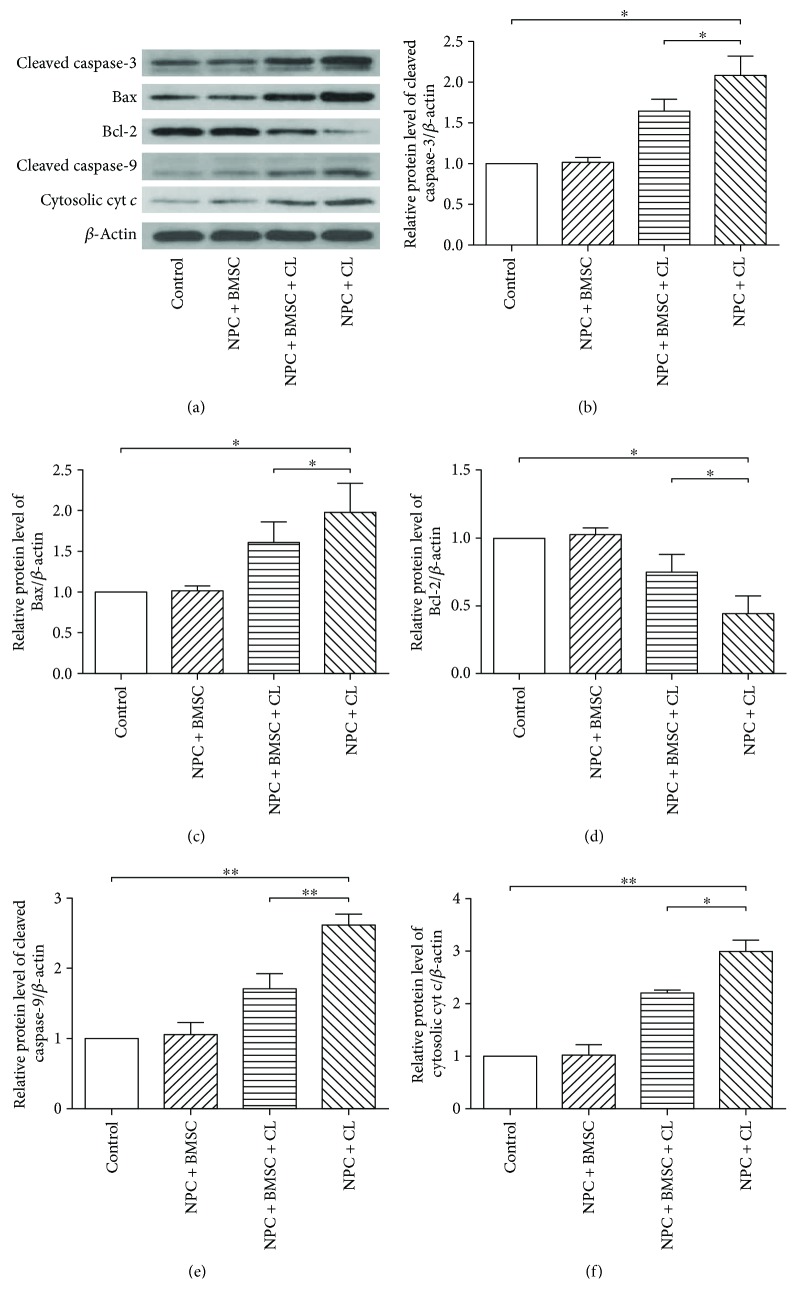
The protein expression of cleaved caspase-3, Bax, Bcl-2, cleaved caspase-9, and cytosolic cytochrome *c* (cytosolic cyt *c*) determined by Western blotting. (a) The typical Western blot bands of cleaved caspase-3, Bax, Bcl-2, cleaved caspase-9, and cytosolic cytochrome *c* (cytosolic cyt *c*). (b), (c), (d), (e), (f) Summary data showing protein levels of cleaved caspase-3, Bax, Bcl-2, cleaved caspase-9, and cytosolic cytochrome *c* (cytosolic cyt *c*). CL means compression load. The data are expressed as mean ± SD from three independent experiments (^∗^*P* < 0.05 and ^∗∗^*P* < 0.01 versus control or NPC + BMSC + CL).

The expression of the mitochondria-mediating proteins (cleaved caspase-3 and-9, cytochrome *c*, Bax, and Bcl-2) was determined by Western blotting. As shown in Figure 6, compression treatment resulted in increased cleaved caspase-3 and -9, cytosolic cytochrome *c*, and Bax and decreased Bcl-2 compared to the control group. However, coculturing with BMSCs partially reversed the changes of the protein level (*P* < 0.05). All these results indicated that BMSCs could protect NPCs from compression-induced apoptosis by inhibiting the mitochondrial or intrinsic pathway.

## 4. Discussion

Currently, there are limited long-lasting and effective treatments in the IVDD therapy. In recent years, many studies have demonstrated that MSCs, especially BMSC-based therapies, are promising for IVD repair [20–22]. In a short-term follow-up of disc cell therapy in vivo, Omlor et al. [23] suggested that BMSCs could keep metabolic activity in a porcine nucleotomy model after 3 days. And in other different degenerative disc models, it was reported that BMSC transplantation could improve the ECM synthesis and disc height [24–26]. These results provide some indication that BMSCs are able to adapt to the degenerative microenvironment and initiate a protective function or an anabolic response, therefore, to regenerate the disc. Apart from BMSCs, other kinds of MSCs, such as adipose-derived MSCs [27] and umbilical cord-derived MSCs [28], could also delay the IVDD.

Although MSCs showed encouraging regeneration effect for IVDD, the mechanism of which is unclear. A lot of studies suggested that the regeneration effect was achieved by the interaction between MSCs and NPCs under different conditions [29]. On one hand, coculturing of MSCs and NPCs promote the differentiation and migration of MSCs [15, 16, 30]. On the other hand, MSCs increase anabolism and decrease catabolism of NPCs and induce an anti-inflammatory effect [15, 17, 31–33]. However, there are few reports discussing the effect of MSC on the NP cell death, such as apoptosis.

In the present study, an indirect coculture system exposed to compression was established to explore the antiapoptosis effect of BMSC on NPCs and the underlying mechanism under compression condition. In our previous studies, we demonstrated that excessive compression would induce NPC apoptosis and result in IVDD [12, 18]. The results in this study were consistent with it and more importantly, we found that BMSCs could protect against compression-induced apoptosis, which was indicated by flow cytometric analysis and morphologic observation.

There are two signaling pathways of apoptosis, mitochondrial (intrinsic) and extrinsic pathways. The mitochondrial pathway had been verified to be involved in various stress-induced apoptosis of NPCs in our previous study and other studies [4, 5, 12]. Therefore, we assumed that the mitochondrial pathway was involved in the antiapoptosis effect of BMSCs on compression-treated NPCs.

ROS are formed primarily from the mitochondria and play an important role in cell signaling and homeostasis in normal physical level. But various stresses, such as compression, can enhance the production of ROS [12]. Excessive ROS can damage mitochondrial function and activate the mitochondrial apoptotic pathway, which manifests as a decrease of MMP and release of cytochrome *c* and then lead to cell apoptosis [34]. Our data showed that compression treatment could significantly increase the ROS level and decrease the MMP, and coculturing with BMSCs partially reversed the change. The protective effect was also confirmed by TEM, which intuitively exhibited the changes of mitochondrial ultrastructure in different groups. These results suggested that BMSCs could protect against compression-induced mitochondrial damage, and the mitochondrial pathway might involve in the antiapoptosis effect.

To further verify our hypothesis at a molecular level, we measured the expression of caspase-3 and -9, cytosolic cytochrome *c*, Bax, and Bcl-2. Bax is a proapoptotic protein, while Bcl-2 is an antiapoptotic protein. Both of them are two classical biomarkers for the mitochondrial pathway and belong to Bcl-2 family proteins [35–37]. In the nondegenerative human lumbar intervertebral disc, Wang et al. [38] reported that there was a high expression of Bcl-2 and a low expression of Bax. Conversely, in the degenerative IVD, Bcl-2 expression was decreased and Bax was increased. And the Bax/Bcl-2 complex dissociation led to the release of cytochrome *c*. Cytochrome *c*, along with Apaf-1 and caspase-9, forms multiprotein apoptosome, which ultimately produces cleaved caspase-9 and -3 and leads to the cell apoptosis [39]. Indeed, an increase of cleaved caspase-3 and -9, upregulation of cytosolic cytochrome *c* and Bax, and downregulation of Bcl-2 was detected in the NPCs with compression treatment compared to the control group, which was in line with our previous study [12]. More importantly, this effect was significantly attenuated by coculturing with BMSCs. Clearly, our findings confirmed that the compression-induced NPC apoptosis was mediated via the mitochondrial apoptotic pathway (Supplementary Figure S1), and the mitochondrial apoptotic pathway was involved in the antiapoptosis effect of BMSCs.

Certainly, there were some limitations of the study. First, the antiapoptosis effect of BMSCs on NPCs was performed in vitro and based on the rat cells. So, further studies with human cells and animal studies need to be carried out. Second, the results in the present study suggested that the compression loading could induce NPC apoptosis through the mitochondrial apoptotic pathway. However, how NPCs sensed compression loading and converted it to apoptotic signals remained unclear. It was reported that transmembrane calcium ion channels, receptor tyrosine kinases, and integrins were the major mechanosensors [8]. Therefore, further studies to identify the mechanosensors are needed (Supplementary Figure S1). Finally, although the results in the present study demonstrated that BMSCs could protect against compression-induced apoptosis of NPCs by inhibiting the mitochondrial pathway, the precise mechanism of the antiapoptosis effect was not fully understood. The use of 0.4 *μ*m pore-size transwell inserts ensured that only secreted factors were easily passed. It indicated that the BMSCs suppressed the apoptosis of NPCs, at least in part, through the paracrine mechanism. It was reported that some mediators (including growth factors, cytokines, chemokines, anti-inflammatory factors, and exosomes) played an essential role in the interaction between MSCs and IVD cells [40–43]. So, what specific secreted factor that plays a major role in the antiapoptosis effect remains to be explored (Supplementary Figure S1).

In conclusion, findings from our study demonstrated the antiapoptosis effect of BMSC on NPCs exposed to compression in vitro. In addition, our data suggested that the mitochondrial apoptotic pathway was involved in the antiapoptosis effect. These results of the present study clarify the underlying molecular mechanism of the antiapoptosis effect and enhance the understanding of the regenerative effect of MSCs.

## Supplementary Materials

Supplementary Materials The schematic diagram of the mechanism and signaling pathway of the antiapoptosis effect of BMSCs on NPCs under compression. Compression loadings enhanced the production of ROS, decreased mitochondrial membrane potential, suppressed the expression of Bcl-2, and increased the expression of Bax. And the Bax/Bcl-2 complex dissociation led to the release of cytochrome *c*. Cytochrome *c*, along with Apaf-1 and caspase-9, formed multiprotein apoptosome, which ultimately produced cleaved caspase-9 and 3 and led to the cell apoptosis. These effects were significantly attenuated by coculturing with BMSCs. The potential mechanosensors of NPCs and paracrine factors secreted by BMSCs remain to be explored.

## References

[1] Rampersaud Y. R., Bidos A., Fanti C., Perruccio A. V. (2017). The need for multidimensional stratification of chronic low back pain (LBP). *Spine*.

[2] Hoy D., Bain C., Williams G. (2012). A systematic review of the global prevalence of low back pain. *Arthritis & Rheumatism*.

[3] Tong W., Lu Z., Qin L. (2017). Cell therapy for the degenerating intervertebral disc. *Translational Research*.

[4] Yang L., Rong Z., Zeng M. (2015). Pyrroloquinoline quinone protects nucleus pulposus cells from hydrogen peroxide-induced apoptosis by inhibiting the mitochondria-mediated pathway. *European Spine Journal*.

[5] Shen J., Xu S., Zhou H. (2017). IL-1β induces apoptosis and autophagy via mitochondria pathway in human degenerative nucleus pulposus cells. *Scientific Reports*.

[6] Jiang W., Zhang X., Hao J. (2015). SIRT1 protects against apoptosis by promoting autophagy in degenerative human disc nucleus pulposus cells. *Scientific Reports*.

[7] Chen D., Xia D., Pan Z. (2016). Metformin protects against apoptosis and senescence in nucleus pulposus cells and ameliorates disc degeneration *in vivo*. *Cell Death & Disease*.

[8] Neidlinger-Wilke C., Galbusera F., Pratsinis H. (2014). Mechanical loading of the intervertebral disc: from the macroscopic to the cellular level. *European Spine Journal*.

[9] Bowles R. D., Setton L. A. (2017). Biomaterials for intervertebral disc regeneration and repair. *Biomaterials*.

[10] Le Maitre C. L., Freemont A. J., Hoyland J. A. (2009). Expression of cartilage-derived morphogenetic protein in human intervertebral discs and its effect on matrix synthesis in degenerate human nucleus pulposus cells. *Arthritis Research & Therapy*.

[11] Sun Z., Luo B., Liu Z. H. (2015). Adipose-derived stromal cells protect intervertebral disc cells in compression: implications for stem cell regenerative disc therapy. *International Journal of Biological Sciences*.

[12] Ding F., Shao Z. W., Yang S. H., Wu Q., Gao F., Xiong L. M. (2012). Role of mitochondrial pathway in compression-induced apoptosis of nucleus pulposus cells. *Apoptosis*.

[13] Longo U. G., Papapietro N., Petrillo S., Franceschetti E., Maffulli N., Denaro V. (2012). Mesenchymal stem cell for prevention and management of intervertebral disc degeneration. *Stem Cells International*.

[14] Oehme D., Goldschlager T., Ghosh P., Rosenfeld J. V., Jenkin G. (2015). Cell-based therapies used to treat lumbar degenerative disc disease: a systematic review of animal studies and human clinical trials. *Stem Cells International*.

[15] Sun Z., Liu Z. H., Zhao X. H. (2013). Impact of direct cell co-cultures on human adipose-derived stromal cells and nucleus pulposus cells. *Journal of Orthopaedic Research*.

[16] Allon A. A., Butcher K., Schneider R. A., Lotz J. C. (2012). Structured coculture of mesenchymal stem cells and disc cells enhances differentiation and proliferation. *Cells, Tissues, Organs*.

[17] Yang S. H., Wu C. C., Shih T. T., Sun Y. H., Lin F. H. (2008). In vitro study on interaction between human nucleus pulposus cells and mesenchymal stem cells through paracrine stimulation. *Spine*.

[18] Ma K. G., Shao Z. W., Yang S. H. (2013). Autophagy is activated in compression-induced cell degeneration and is mediated by reactive oxygen species in nucleus pulposus cells exposed to compression. *Osteoarthritis and Cartilage*.

[19] Kong D., Zhu J., Liu Q. (2017). Mesenchymal stem cells protect neurons against hypoxic-ischemic injury via inhibiting parthanatos, necroptosis, and apoptosis, but not autophagy. *Cellular and Molecular Neurobiology*.

[20] Huang Y., Leung V. Y. L., Lu W. W., Luk K. D. (2013). The effects of microenvironment in mesenchymal stem cell–based regeneration of intervertebral disc. *The Spine Journal*.

[21] Leung V. Y. L., Aladin D. M. K., Lv F. (2014). Mesenchymal stem cells reduce intervertebral disc fibrosis and facilitate repair. *Stem Cells*.

[22] Zhao Y., Jia Z., Huang S. (2017). Age-related changes in nucleus pulposus mesenchymal stem cells: an in vitro study in rats. *Stem Cells International*.

[23] Omlor G. W., Fischer J., Kleinschmitt K. (2014). Short-term follow-up of disc cell therapy in a porcine nucleotomy model with an albumin-hyaluronan hydrogel: in vivo and in vitro results of metabolic disc cell activity and implant distribution. *European Spine Journal*.

[24] Yang F., Leung V. Y., Luk K. D., Chan D., Cheung K. M. (2009). Mesenchymal stem cells arrest intervertebral disc degeneration through chondrocytic differentiation and stimulation of endogenous cells. *Molecular Therapy*.

[25] Yang H., Wu J., Liu J. (2010). Transplanted mesenchymal stem cells with pure fibrinous gelatin-transforming growth factor-β1 decrease rabbit intervertebral disc degeneration. *The Spine Journal*.

[26] Zhang Y., Drapeau S., Howard S. A., Thonar E. J. M. A., Anderson D. G. (2011). Transplantation of goat bone marrow stromal cells to the degenerating intervertebral disc in a goat disc injury model. *Spine*.

[27] Marfia G., Campanella R., Navone S. E. (2014). Potential use of human adipose mesenchymal stromal cells for intervertebral disc regeneration: a preliminary study on biglycan-deficient murine model of chronic disc degeneration. *Arthritis Research & Therapy*.

[28] Pang X., Yang H., Peng B. (2014). Human umbilical cord mesenchymal stem cell transplantation for the treatment of chronic discogenic low back pain. *Pain Physician*.

[29] Richardson S. M., Kalamegam G., Pushparaj P. N. (2016). Mesenchymal stem cells in regenerative medicine: focus on articular cartilage and intervertebral disc regeneration. *Methods*.

[30] Hu X., Zhou Y., Zheng X. (2014). Differentiation of menstrual blood-derived stem cells toward nucleus pulposus-like cells in a coculture system with nucleus pulposus cells. *Spine*.

[31] Ouyang A., Cerchiari A. E., Tang X. (2016). Effects of cell type and configuration on anabolic and catabolic activity in 3D co-culture of mesenchymal stem cells and nucleus pulposus cells. *Journal of Orthopaedic Research*.

[32] Cao C., Zou J., Liu X. (2015). Bone marrow mesenchymal stem cells slow intervertebral disc degeneration through the NF-ĸB pathway. *The Spine Journal*.

[33] Yang H., Cao C., Wu C. (2015). TGF-βl suppresses inflammation in cell therapy for intervertebral disc degeneration. *Scientific Reports*.

[34] Zhang C. X., Wang T., Ma J. F., Liu Y., Zhou Z. G., Wang D. C. (2017). Protective effect of CDDO-ethyl amide against high-glucose-induced oxidative injury via the Nrf2/HO-1 pathway. *The Spine Journal*.

[35] Chen J. H., Yang C. H., Wang Y. S., Lee J. G., Cheng C. H., Chou C. C. (2013). Acrylamide-induced mitochondria collapse and apoptosis in human astrocytoma cells. *Food and Chemical Toxicology*.

[36] Kim K. W., Ha K. Y., Lee J. S., Rhyu K. W., An H. S., Woo Y. K. (2007). The apoptotic effects of oxidative stress and antiapoptotic effects of caspase inhibitors on rat notochordal cells. *Spine*.

[37] Nguyen K. C., Willmore W. G., Tayabali A. F. (2013). Cadmium telluride quantum dots cause oxidative stress leading to extrinsic and intrinsic apoptosis in hepatocellular carcinoma HepG2 cells. *Toxicology*.

[38] Wang H., Liu H., Zheng Z. M. (2011). Role of death receptor, mitochondrial and endoplasmic reticulum pathways in different stages of degenerative human lumbar disc. *Apoptosis*.

[39] Sinha K., Das J., Pal P. B., Sil P. C. (2013). Oxidative stress: the mitochondria-dependent and mitochondria-independent pathways of apoptosis. *Archives of Toxicology*.

[40] Sakai D., Grad S. (2015). Advancing the cellular and molecular therapy for intervertebral disc disease. *Advanced Drug Delivery Reviews*.

[41] Fontana G., See E., Pandit A. (2015). Current trends in biologics delivery to restore intervertebral disc anabolism. *Advanced Drug Delivery Reviews*.

[42] Lu K., Li H. Y., Yang K. (2017). Exosomes as potential alternatives to stem cell therapy for intervertebral disc degeneration: in-vitro study on exosomes in interaction of nucleus pulposus cells and bone marrow mesenchymal stem cells. *Stem Cell Research & Therapy*.

[43] Cheng X., Zhang G., Zhang L. (2017). Mesenchymal stem cells deliver exogenous miR-21 *via* exosomes to inhibit nucleus pulposus cell apoptosis and reduce intervertebral disc degeneration. *Journal of Cellular and Molecular Medicine*.

