# Supplementary material for: Mesenchymal Stem Cells Protect Nucleus Pulposus Cells from Compression-Induced Apoptosis by Inhibiting the Mitochondrial Pathway
Source: Stem Cells Int. 2017 Dec 14;2017:9843120. doi: 10.1155/2017/9843120 (PMC5745742; doi:10.1155/2017/9843120)
Supplement: Supplementary Materials — The schematic diagram of the mechanism and signaling pathway of the antiapoptosis effect of BMSCs on NPCs under compression. Compression loadings enhanced the production of ROS, decreased mitochondrial membrane potential, suppressed the expression of Bcl-2, and increased the expression of Bax. And the Bax/Bcl-2 complex dissociation led to the release of cytochrome c. Cytochrome c, along with Apaf-1 and caspase-9, formed multiprotein apoptosome, which ultimately produced cleaved caspase-9 and 3 and led to the cell apoptosis. These effects were significantly attenuated by coculturing with BMSCs. The potential mechanosensors of NPCs and paracrine factors secreted by BMSCs remain to be explored. [file 9843120.f1.pptx]

## Slide 1
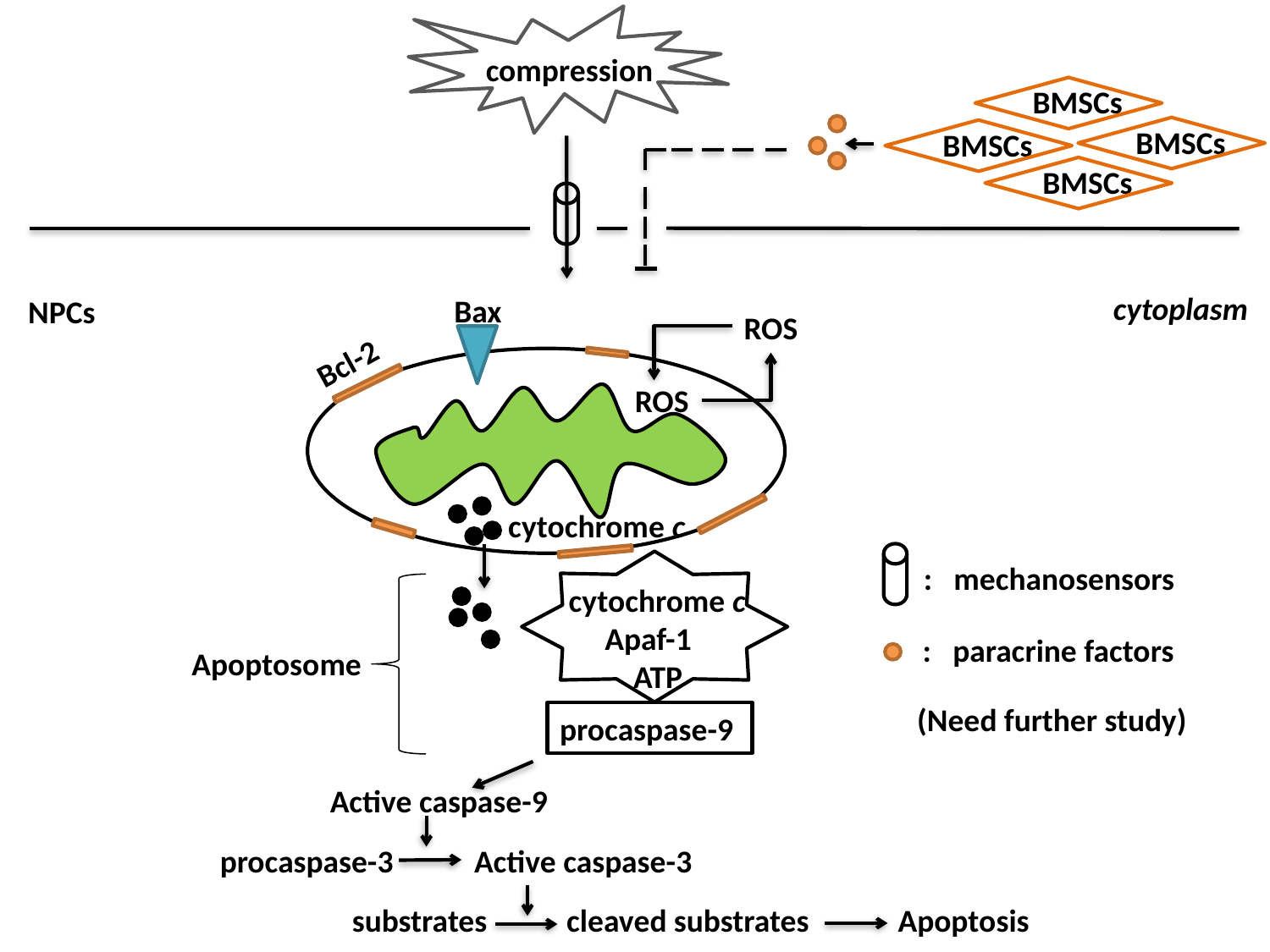

compression
BMSCs
BMSCs
BMSCs
BMSCs
cytoplasm
Bax
NPCs
ROS
Bcl-2
ROS
cytochrome c
: mechanosensors
cytochrome c
 Apaf-1
 ATP
: paracrine factors
Apoptosome
(Need further study)
procaspase-9
Active caspase-9
procaspase-3
Active caspase-3
substrates
cleaved substrates
Apoptosis

## Slide 2
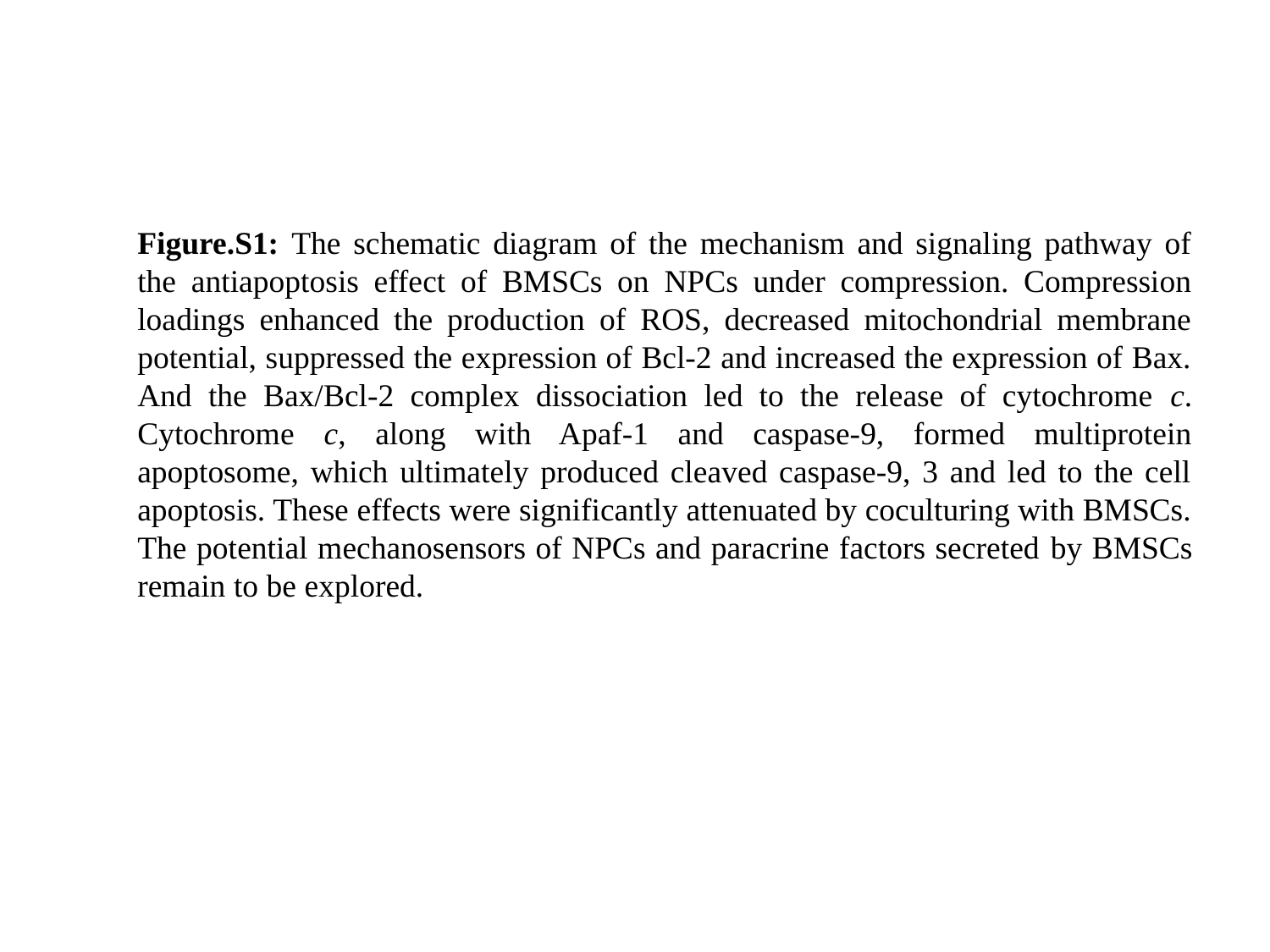

Figure.S1: The schematic diagram of the mechanism and signaling pathway of the antiapoptosis effect of BMSCs on NPCs under compression. Compression loadings enhanced the production of ROS, decreased mitochondrial membrane potential, suppressed the expression of Bcl-2 and increased the expression of Bax. And the Bax/Bcl-2 complex dissociation led to the release of cytochrome c. Cytochrome c, along with Apaf-1 and caspase-9, formed multiprotein apoptosome, which ultimately produced cleaved caspase-9, 3 and led to the cell apoptosis. These effects were significantly attenuated by coculturing with BMSCs. The potential mechanosensors of NPCs and paracrine factors secreted by BMSCs remain to be explored.
